# Cognitive analytic therapy‐guided self‐help for depression: A mixed methods evaluation

**DOI:** 10.1111/papt.70008

**Published:** 2025-08-18

**Authors:** Rebecca Kelly, Stephen Kellett, Mel Simmonds‐Buckley, Niall Power

**Affiliations:** ^1^ Rotherham Doncaster and South Humber NHS Foundation Trust, South Yorkshire UK; ^2^ University of Sheffield, Sheffield UK; ^3^ University of Exeter Exeter UK; ^4^ South West Yorkshire Partnership NHS Foundation Trust, South Yorkshire UK

**Keywords:** cognitive analytic therapy, guided self‐help, IAPT

## Abstract

**Objectives:**

To evaluate cognitive analytic therapy‐guided self‐help (CAT‐GSH) for depression in terms of outcomes and acceptability.

**Design:**

A parallel mixed methods case series with session‐by‐session outcome measurement supplemented by patient and staff interviewing.

**Methods:**

Four psychological well‐being practitioners (PWPs) delivered CAT‐GSH to *N* = 11 patients in an NHS talking therapies service, and these patients were followed up at 1 month. Acceptability of CAT‐GSH was assessed via treatment refusal and dropout rates, sessional attendance and qualitative interviewing of PWPs and patients. Outcomes were assessed by comparing group means at screening, termination and follow‐up on sessional measures, calculating the case‐by‐case recovery rate and benchmarking against relevant research.

**Results:**

All 11 patients offered CAT‐GSH accepted; one patient dropped out, all completers attended the full 6 sessions, and 7/10 were in reliable recovery at follow‐up. Treatment gains were maintained over the follow‐up period, and outcomes appeared equivalent when benchmarked against the evidence base. Patients found CAT‐GSH to be a mostly acceptable intervention, and this was due to mood improvements, better recognition skills and implementation of ‘exits’. CAT‐GSH improved the diversity of the treatment offer for PWPs and provided professional development opportunities.

**Conclusions:**

CAT‐GSH holds promise as a low‐intensity treatment for depression, but clearly more controlled research is needed.

## INTRODUCTION

In NHS talking therapies (TT) for anxiety and depression (previously known as improving access to psychological therapies, IAPT) psychological well‐being practitioners (PWPs) deliver low‐intensity cognitive behavioural therapy (LI‐CBT) interventions to patients presenting with mild‐to‐moderate depression (National Institute for Health and Care Excellence; NICE, 2022). LI‐CBT interventions outperform passive controls (Santoft et al., 2019) and the medium by which the intervention is delivered (i.e., individual, group or telephone) does not appear to influence outcomes (Cuijpers et al., 2010). In TT services, 47.9% of patients receiving LI‐CBT ‘reliably recover’ but approximately 45% of referrals drop out (NHS England, 2024a). Approximately 53% of patients completing LI‐CBT relapse within 1 year (Ali et al., 2017), and there is a 13.7% treatment return rate (Lorimer et al., 2024). While LI interventions within TT services are clearly beneficial for some, there is certainly a lack of patient choice regarding type of LI treatment and some evidence of poor acceptability and durability.

To address the lack of LI treatment choice, cognitive analytic therapy (CAT) has been adapted into a guided self‐help format (CAT‐GSH). This has been designed to be delivered by PWPs in a structured 6‐session format, with the patient workbook being mapped onto the three‐phase theoretical structure of CAT (i.e., reformulation, recognition and revision; Ryle & Kerr, 2020). So far, this development and associated evaluation work has been limited to anxiety. In the initial pilot (Meadows & Kellett, 2017), 10/11 patients who attended their first treatment session then completed full treatment, and 6/10 were in reliable recovery at follow‐up. Wray et al. (2022) further investigated treatment acceptability with PWPs and reported increased treatment choice, collaborative therapeutic relationships, increased insight and concrete changes, but also negatively that PWPs initially lacked confidence. Kellett et al. (2023) conducted a large (*N* = 271) patient preference trial of CAT‐GSH versus CBT‐GSH. Both interventions were competently delivered, and there were reductions in anxiety over time for participants in both arms, but no differences were found between arms. CAT‐GSH was more preferred (72% vs. 28%) and CAT‐GSH participants were significantly more likely to start GSH, attend all sessions and attend significantly more sessions overall. When the change process between CAT‐GSH versus CBT‐GSH has been compared, CAT‐GSH facilitates relational insight and change, whereas CBT‐GSH facilitates better knowledge, coping and more supportive relationships (Headley et al., 2024).

There is now a need to develop and evaluate CAT‐GSH for depression to mirror the anxiety evidence base. While CAT uses a transdiagnostic approach (Balmain et al., 2021) and the three‐phase structure would remain (Meadows & Kellett, 2017), the CAT‐GSH needed to be redesigned with a depression focus to ensure face validity for those presenting with depression. This innovation was also informed by the Medical Research Council (Craig et al., 2008) treatment development guidelines. The current study therefore reports a mixed methods evaluation of the acceptability and outcomes produced by CAT‐GSH for depression. The outcomes of an intervention delivered in routine practice are the domain of effectiveness studies, whereas during randomised controlled trials (RCTs), the efficacy of an intervention is assessed under controlled experimental conditions (Rosqvist et al., 2011). Therefore, the current study would be an example of early practice‐based research (Castonguay et al., 2021), where CAT‐GSH for depression is delivered in a real‐world setting, with few exclusion criteria and no randomisation. Whilst practice‐based studies tend not to measure treatment integrity (Rosqvist et al., 2011), the current study also sought to assess treatment competency to increase the interval validity of the study.

Mixed methods were chosen for the current study because the CAT‐GSH evidence base has previously neglected collecting qualitative patient views on acceptability. Better understanding the patient experience could help to usefully adjust CAT‐GSH for depression and consider how the GSH can then best be delivered in routine practice for this patient group (Chenail, 2011). In terms of aims, the current study sought to explore the promise of CAT‐GSH for depression as a service development. Due to the practice‐based design, it is impossible to confidently attribute any outcomes observed to CAT‐GSH for depression delivered at this stage. Specifically, the study assessed acceptability by measuring the treatment refusal and treatment dropout rate and set a progression target of how many patients would need to be treated to then create a case series with *n* = 10 fully completed (i.e., 6‐session) CAT‐GSH treatments. NHS England has recently set a target for TT services that 48% of eligible referrals should move to reliable recovery (NHS England, 2024b); therefore, the CAT‐GSH recovery rate was assessed against this target.

## METHOD

### Ethical approval and design

Ethical approval was obtained (registration number: 310527). A parallel mixed methods case series design was the methodology for the study. Acceptability was measured quantifiably by measuring the treatment refusal rate (i.e., number of participants offered CAT that never then attended for treatment), the treatment dropout rate and the session attendance rate. Acceptability was measured qualitatively using the theoretical framework of acceptability (TFA; Sekhon et al., 2017) of affective attitude, burden, ethicality, intervention coherence, opportunity costs, perceived effectiveness and self‐efficacy. Outcomes were assessed on sessional clinical outcome measures at both a group level and on a case‐by‐case basis. A critical realist‐informed perspective helped develop and implement this research. It was assumed that the reality of CAT‐GSH was impacted by the researchers, PWPs and patients' perspectives alongside broader social narratives and the influence of wider healthcare systems (Stutchbury, 2021; Zhang, 2023). A subjective epistemological position assumed that the measurement of the reality was impacted by personal beliefs, interpretations and measurement constructs.

### Participants

Four qualified PWPs working in TT were recruited using opportunity sampling. Recruited PWPs screened the LI treatment waiting list and offered CAT‐GSH to patients when (a) depression was the main problem, (b) the patient scored above the clinical cut‐off (>10) on the Patient Health Questionnaire‐9, (c) was aged between 18 and 65, (d) would prefer to use CAT‐GSH over other interventions, (e) consented to participate in the study and (f) could engage with the written psychoeducational workbook. The exclusion criteria were (a) patients who needed to be ‘stepped up’ due to complexity and risk, (b) patients receiving other therapies and (c) substance users that were unable to abstain during the GSH. Due to human error, one participant received CAT‐GSH but was below PHQ‐9 caseness at screening.

### Procedure

PWPs attended a 2‐day training course facilitated by the researchers. The content included specifics of CAT, working with the manual and research processes. PWPs completed an 8‐item training satisfaction questionnaire, and Supporting Information report these scores. The highest frequency of words to describe training included interesting, informative and thorough. Monthly group clinical supervision was provided to the PWPs alongside their regular, individual, weekly case management supervision as normal in the TT service.

### Intervention and competency

CAT‐GSH was delivered on a weekly basis and provided face‐to‐face or via video link, and sessions were 35 min in duration. Participants received a printed copy of the GSH manual. Session three was recorded to assess PWP competency. Two members of the research team scored the PWPs with the validated low‐intensity treatment competency scale (LITC; Kellett et al., 2021). Raters were the lead researcher (RK) and a clinical psychologist (NP). The second rater had been the primary rater for the competency study for the CAT‐GSH patient prefernce trial (Power et al., 2024) and trained RK for the current study in terms of accurately using the competency measure. A total score of 18 or above is defined as reaching GSH clinical competence. Competency is reported by providing each PWPs score, the rate at which competency CAT‐GSH was apparent (session scores >18) and the mean competency score for the sample. The inter‐rater reliability, calculated using intraclass correlation coefficient, was 95%.

### Outcome measures

The self‐report measures listed below were collected at screening, each treatment session and at 1‐month follow‐up.

#### Patient Health Questionnaire‐9 (PHQ‐9)

This scale identifies depression and is based on the DSM‐IV criteria (Kroenke et al., 2001). It has a clinical cut‐off of >10, and when the score reduces by 6 or more, then this is a reliable reduction. Sensitivity and specificity have been identified at 88% and 85%, respectively, at the >10 cut‐off point (Levis et al., 2019). The internal reliability of the PHQ‐9 is good (*α* = .89; Kroenke et al., 2001).

#### Generalised Anxiety Disorder‐7 (GAD‐7)

The GAD‐7 (Spitzer et al., 2006) identifies anxiety and has a clinical cut‐off >8. When the GAD‐7 score reduces by 4 or more, then this is a reliable reduction in anxiety. The GAD‐7 has a sensitivity of 83% and specificity of 84% when applying a threshold score of 8 (Plummer et al., 2016). The internal reliability of the GAD‐7 is good (*α* = .88; Johnson et al., 2019).

#### Work and Social Adjustment Scale (WSAS)

The WSAS (Mundt et al., 2002) measures social functioning. It has a clinical cut‐off of >10, is sensitive to treatment effects and has good internal reliability (*α* = .82; Zahra et al., 2014). The WSAS has a sensitivity of 74% and a specificity of 55% in the prediction of sick‐leave status (Hovmand et al., 2024).

### Outcome definitions and analyses

Rates of reliable and clinically significant change rates were comparedon screening to termination scores and screening to follow‐up scores. PHQ‐9 scores for each participant were categorized as follows: (1) uncertain change, where change was unreliable; (2) reliable deterioration, where scores reliably worsened; (3) reliable improvement, where scores reliably improved and (4) reliable recovery, where scores reliably improved, and scores shifted from above clinical cut‐off pre‐treatment to below clinical cut‐off post‐treatment. Sessional medians on the three outcome measures are graphed, and a plot of PHQ‐9 sessional outcomes with associated confidence intervals is provided. Treatment characteristics, uptake rates, dropout rates and group level means and SDs for screening, termination and follow‐up scores on each outcome measure are reported in the benchmarking exercise.

### Qualitative interviews and analysis

Individual semi‐structured exit interviews (in person and online by preference) with PWPs and patients that completed CAT‐GSH for depression were completed by the lead researcher and were transcribed by an approved party. The interviewer did not deliver any of the GSH interventions. A reflexivity log was created pre‐ and post‐interviews. Framework analysis (FA; Ritchie & Spencer, 1994) analysed the exit interview data and was completed by the lead researcher. FA is not bound by a specific epistemological position and provides‘best fit’ for this type of research (Gale et al., 2013; Ritchie & Spencer, 1994). PWP and patient data were analysed together to incorporate the shared experiences of the professional and the patient. Familiarization included listening to audio recordings alongside rereading the reflexivity logs and transcripts. Familiarization notes made from the process reflected the TFA model. From this process, an initial framework was created and tested on two PWP and three patient transcripts. Revisions to the framework were made, and once the final framework was outlined, all interviews were indexed using NVIVO software. The indexes were formatted into a theme‐organized chart; this matrix was mapped, and interpretations were made to explore the acceptability of CAT‐GSH. Auditing was completed at the end of this process. The quality assurance, reflexivity and ontological position statements are included in Supporting Information.

## RESULTS

### Acceptability

*N* = 98 patients on the waiting list for a step 2 intervention were screened for suitability. Twelve (12.24%) were deemed potentially suitable for CAT‐GSH for depression, *n* = 80 did not have depression as a presenting problem, *n* = 5 did not meet the inclusion criteria and *n* = 1 requested discharge. Of the 12 eligible patients, one was unable to coordinate diaries with the PWP providing CAT‐GSH and therefore completed CBT‐GSH with another PWP. Of the *n* = 11 offered CAT‐GSH, 11 then accepted (i.e., the treatment refusal rate = zero). Ten completed CAT‐GSH and the follow‐up (i.e., the ‘treatment completer group’). One participant dropped out at the third session. In the completer group, all CAT‐GSH sessions were attended. Demographics and attendance are reported in Table 1. These are summarised as the patient age ranging from 18 to 59 years (mean = 42.45) with two male and three female patients.

**TABLE 1:** Demographics, session frequency and attendance.

	Gender	Age	Session frequency	Session attendance
P1	Female	55	Weekly	6/6
P2	Female	39	Weekly	6/6
P3	Female	53	Weekly	6/6
P4	Female	40	Weekly	6/6
P5	Female	18	Weekly	3/6
P6	Male	59	Weekly	6/6
P7	Male	29	Weekly	6/6
P8	Female	29	Weekly	6/6
P9	Female	49	Weekly	6/6
P10	Male	41	Weekly	6/6
P11	Female	55	Weekly	6/6

*Note*: P5 dropped out after Session 3.

### Competency

Session competency ratings are reported in Table 2. The overall mean (18.70) was above the cut‐off score (>18) and 6/10 sessions were above the competency cut‐off score.

**TABLE 2:** Overall competency scores, means and competency criteria rates.

	Rater one	Rater two	Mean	Competency criteria met (score >18)
P1	19	19	19	Yes
P2	18	18	18	Yes
P3	17.5	18	17.75	No
P4	16	17	16.5	No
P5	22.5	22.5	22.5	Yes
P6	23	21	22	Yes
P7	21	21	21	Yes
P8	16	18	17	No
P9	18.5	19.5	19	Yes
P10	16.5	16.5	16.5	No
P11	15	16	15.5	No
Overall mean (*SD*)	18.45 (2.70)	18.95 (2.05)	–	–

### Group‐level outcomes

**FIGURE 1:**
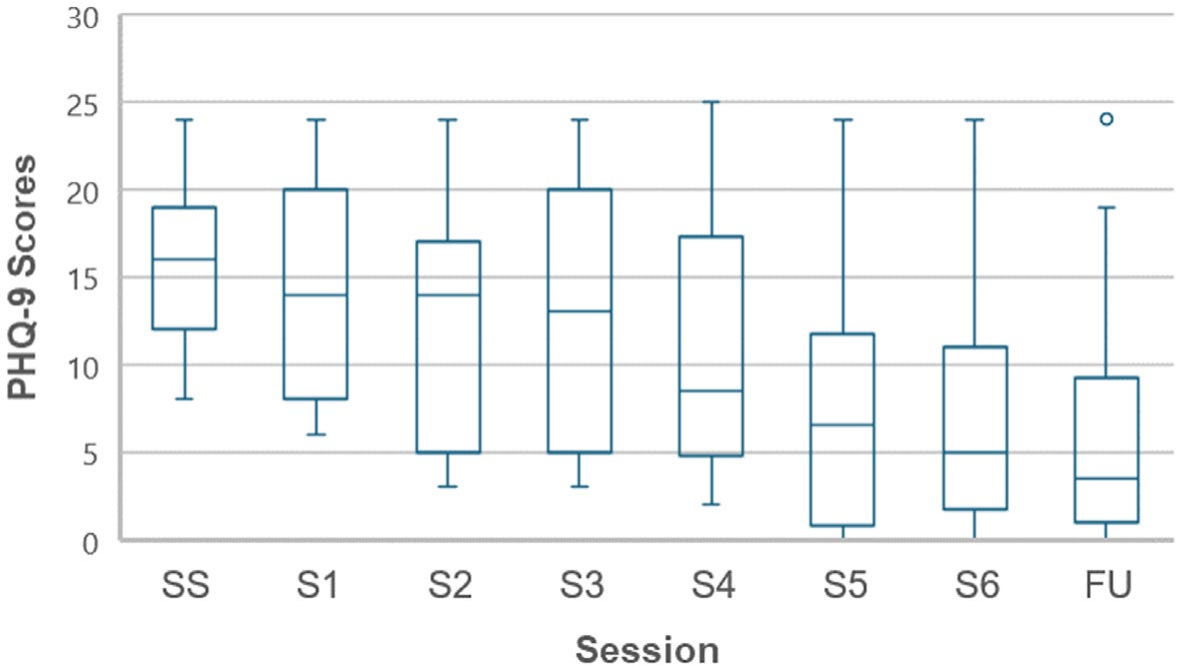
Distribution of group PHQ‐9 scores across 6 sessions and follow‐up.

**FIGURE 2:**
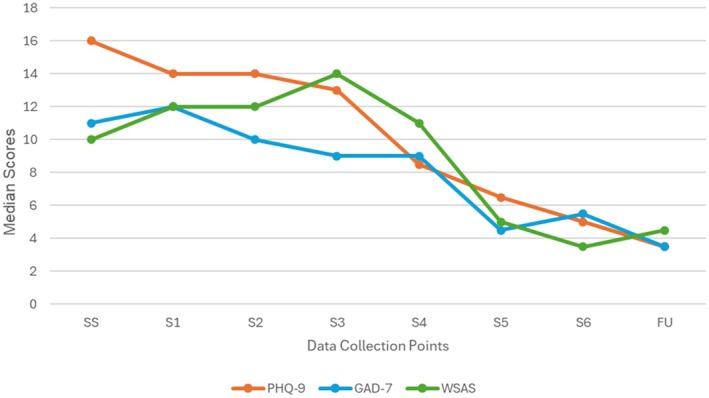
Median group scores for the PHQ‐9, GAD‐7 and WSAS over sessional time.

Figure 1 contains a box and whisker chart demonstrating the distribution of the PHQ‐9 scores at screening, across each of the CAT‐GSH sessions and follow‐up, with confidence intervals around sessional means. Figure 2 presents median group scores across screening, treatment sessions and the follow‐up for all three of the outcome measures. These figures suggest symptoms and disability reduce over time during CAT‐GSH and that follow‐up scores tend to overlap with end of treatment scores. Table 3 contextualizes these outcomes by providing benchmarks for CAT‐GSH and CBT‐GSH to suggest equivalence with the evidence base.

**TABLE 3:** Benchmark outcomes compared to present study.

Author	Presenting problem (sample size)	Treatment	Uptake (%)	Attrition (%)	Outcome measure	Outcome			
						Screening session mean (*SD*)	Termination mean (*SD*)	Follow‐up mean (*SD*)	Change score (S–T)
Present study	Depression (*n* = 11)	CAT‐GSH, screening session, 6 × 35 min sessions face‐to‐face or via Microsoft Teams	100	9.09	PHQ‐9 GAD‐7 WSAS	16.36 (4.84) 11.73 (5.41) 11 (5.6)	8.27 (7.91) 8 (6.54) 9.09 (9.61)	6.4 (8.28) 5.8 (6.91) 7.5 (9.9)	−8.27 −3.73 −1.91
Green et al. (2014)	Depression and/or anxiety (*n* = 1122)	GSH within NHS talking therapies, screening plus 2–21 face‐to‐face sessions	–	–	PHQ‐9 GAD‐7	13.7 (6.43) 12.04 (5.57)	9.83 (7.15) 8.99 (6.32)	– –	−3.34 (6.43) −3.05 (5.82)
Kellett et al. (2023)	Anxiety (*n* = 79 partook in CBT‐GSH)	CBT‐GSH, screening plus 6–8 sessions via telephone	–	–	PHQ‐9 GAD‐7 WSAS	16.87 (5.01) 16.86 (3.44) 21.35 (7.41)	– – –	– – –	−4.81 (0.68) −5.84 (0.62) −4.67 (1.06)
Meadows and Kellett (2017)	Anxiety (*n* = 17)	CAT‐GSH, screening session, 6 × 35 min face‐to‐face sessions	42	9	PHQ‐9 GAD‐7 WSAS	12.4 (3.78) 13.2 (3.43) 15.3 (5.08)	7.6 (4.81) 7.5 (4.93) 8.8 (3.65)	5.5 (4.48) 6.3 (4.74) 6.6 (4.77)	−5.7 −4.8 −6.5

### Recovery rates

Supporting Information contain the case‐by‐case outcomes including raw scores at screening, termination and follow‐up and whether reliable and clinically significant change had occurred. The screening to follow‐up outcome categorizations were as follows: 7/11 met reliably recovery, 2/11 had a reliable improvement, and 2/11 had no change.

### Qualitative acceptability outcomes

Overall, CAT‐GSH was experienced as largely acceptable by the patients and the PWPs. All seven core themes of the TFA (Sekhon et al., 2017) were covered and were supported by 16 subthemes.

#### Affective attitude: Positively changing opinions

This theme, supported by 10 patients and 4 PWPs, concerned how individuals felt about CAT‐GSH, and there was evidence that this changed positively over time.

##### Mixed feelings prior to starting CAT‐GSH

PWPs and patients spoke of mixed feelings before starting CAT‐GSH. Some patients were nervous about starting the intervention, while others felt intrigued by what CAT‐GSH could offer. P1 expressed ‘I think from way I were feeling to like actually going in for first session was like “argh” the dread the felt the anxiety of actually I've got to go to that place’. PWPs reported feeling nervous, sceptical and/or intrigued about using CAT‐GSH: ‘I were a little bit sceptical about it to start with’ (PWP1). PWPs were initially unsure what to expect and how CAT‐GSH could help people, but the possibilities of learning a new LI treatment were intriguing.

##### Largely positive feelings

PWPs and patients spoke positively about CAT‐GSH for depression as they reflected on how they felt during and after delivering and receiving the intervention: ‘Erm, no, just that I really enjoyed it, it really helped’. (Patient 2). Patients variously described CAT‐GSH as ‘fantastic, powerful, and enthusiastic’ and attributed these feelings also to the support offered by PWPs. PWPs shared similar sentiments and reported a sense of excitement about CAT‐GSH as an LI intervention. For instance, PWP 3 expressed: ‘No (laughs) I wanted to like, you know, give you something but, no, as you can tell I had a good experience with it so I don't know what I would change to be honest’. However, some PWPs felt anxious when delivering CAT‐GSH and this was attributed to the novelty of the approach and how it differed from CBT‐GSH. For example, some PWPs were unfamiliar with discussing the relationship between themselves and the patient, as prompted throughout the GSH manual.

#### Burden

Burden refers to the perceived amount of effort that is required to participate in CAT‐GSH for depression. The theme was supported by eight participants (i.e., four patients and four PWPs). Common themes included the emotional burden and the effort the PWPs needed to complete the training and preparation.

##### Emotional burden

Four patients described how emotionally taxing it was to complete CAT‐GSH for depression, with the most difficult aspects being speaking about past relationships and the strain of increasing their insight of patterns. Patient 8 stated ‘I managed to find the way to get out of it so but yeah it were a bit doom and gloom like going really root of the problem do you know what I mean?’ However, these experiences were also described as eventually helpful and were attributed to the interventions perceived helpfulness. PWPs also agreed some patients found CAT‐GSH burdensome as it invoked previously avoided emotions, as patients were discussing the origins of their depression. PWP2 expressed ‘I think for some clients, erm, and can be quite I suppose hard hitting, erm, kind of thinking about family and where things might have come from in relationship roles’. One PWP also found the GSH burdensome due to the pressure to deliver CAT‐GSH alongside a caseload of other LI interventions.

##### PWP efforts

Two PWPs expressed that facilitating CAT‐GSH for depression was effortful because of their commitment to other projects (i.e., a long‐term conditions pathway and training to become a CBT therapist). Part of the effort came from learning required to ensure its competent delivery of the GSH and the effort also of switching interventions between patients. PWP 2 expressed: ‘I think the, erm, biggest of the spinning plates of like, getting, trying to get this, getting my head into that and then going to maybe low intensity as usual step two’. One PWP reported that they felt that CAT‐GSH exerted a similar drain of energy and time as delivering CBT‐GSH.

#### Intervention coherence

Coherence is the extent to which the participant understands how CAT‐GSH works. This theme was supported by all 14 participants. A common pattern between the themes was that no one participant described entirely how CAT‐GSH for depression worked. However, participants seemed to understand the aspects that were important to them. Patient and PWPs level of intervention coherence also differentiated, with PWPs having more knowledge.

##### Understanding developed

PWPs and patients explained that the CAT‐GSH for depression manual was initially difficult to understand, however, this did reduce with practice and time. Patients attributed this development of understanding to the support offered by PWPs. PWPs attributed practice, training and supervision to the development of their understanding. For example, PWP4 expressed ‘I'd like more time I think to get to grips with it because obviously it's quite speedy, you know, we were doing that a day of training and that we're kind of in and doing it’.

##### Different parts understood

PWPs reported understanding the theoretical underpinning of CAT‐GSH for depression. PWPs typically demonstrated understanding of each of the three stages of CAT (reformulation, recognition and revision) and how different strategies related to this (e.g., how the family tree helped patients reformulate their depression). Patients' comprehension varied and tended to focus on one aspect of CAT‐GSH (e.g., a sole focus on exit strategies). This may have been related to what the patients found most interesting or valuable. ‘It were, you know, to help me, you know, like with relationships and me, you know, to towards people and why, you know, like why, why I'm a people pleaser and, erm, and I get dismissed and excluded and things but I learnt that it's like it is some things from me, erm, you know like me past which weren't, do you know what I mean’. (Patient 3).

##### CAT language

Both PWPs and patients spoke about their experiences using CAT‐informed terminology and that this terminology was novel information that took some time to learn for the PWPs. This included using terms such as trap, snag, dilemma, exit strategies and relationship roles. The PWPs and patients used CAT terminology alongside more traditional CBT‐informed terminology such as thought challenging and behavioural activation. Patient 11 expressed ‘I mean obviously, erm, there were some of the tips regarding the exit strategies, you know, if you start to get into the downward spiral of like thinking and ruminating 'cos ruminating was one of my biggest problems’.

#### Opportunity costs

This included the extent to which other benefits, profits or values were attained or sacrificed by engaging in CAT‐GSH for depression. The theme was supported by 12 participants (i.e., 8 patients and 4 PWPs). Patients and PWPs generally felt that they had to sacrifice little for CAT‐GSH but also provided examples of compromise. This may reflect that participants found the ‘costs’ of CAT‐GSH were worthwhile.

##### No drawbacks or costs

Patients and PWPs reported they had not had to give up anything to be able to engage with CAT‐GSH for depression. In fact, patients were happy with the flexibility of the PWPs and service which were accommodating to their choice of time and day for sessions to be delivered. Patient 10 explained ‘Everything's been free, and every step of the way has been fantastic, so I can't, there's no complaints of cost or anything like that’. While PWP3 expressed ‘No I didn't have to kind of make any adaptations to be honest really or make any changes it fit exactly as it, as it would do with kind of my standardised PWP work’.

##### Travel and time

Seven of the nine participants who contributed to this subtheme also acknowledge there were opportunity costs, but overall, these were worthwhile sacrifices. For patients this usually involved paying for transport or requesting leave from work: ‘Just mostly because of travel oh God I could do without driving there today’ (Patient 9). For PWPs, they had to make some changes to their work schedules, but this was no more than in their CBT‐GSH work. Furthermore, the service accommodated for the extra supervision. PWP4 stated ‘I had to change my treatments around, erm, to do that, erm, but yeah and just have a make time for supervisions and stuff like that but again within service they allowed us to do that’.

#### Perceived effectiveness

Perceived effectiveness (supported by 14 participants) explored the extent to which the CAT‐GSH for depression was perceived as helpful. PWPs and patients provided information about what aspects of CAT‐GSH contributed to helpfulness alongside providing recommendations for changes.

##### Varied effectiveness

Most patients felt that CAT‐GSH for depression was useful or a step towards recovery. Treatment helped to reduce distress, achieve idiosyncratic goals, improve recognition and build confidence. PWPs stated CAT‐GSH was helpful in terms of improving low mood but also reducing anxiety and anger. ‘I'm, as a therapist, more looking to get people into recovery but that's very subjective 'cos he might not have got into recovery on the questionnaires, but he might have read towards progress in terms of his life in terms of what he wants to get out of it’ (PWP 2). Regarding recovery, Patient 6 expressed ‘I do have my moments still but like I say I'm trying to reverse nearly 60 years of behaviour, but I do feel I recognise things sooner and I do I have I am calm’. Of those who found CAT‐GSH to be unhelpful, one patient explained they continued to be depressed because of ongoing life events that the GSH could not resolve. One PWP also reported using CAT‐GSH for depression to improve their own mood.

#### Helpful and unhelpful components

The most common helpful components of CAT‐GSH for depression for patients were receiving PWPs support, considering the origins of their depression, exploring relationship roles/patterns, as well as finding exit strategies. Patient 6 expressed ‘I don't think there was anything really like, from my experience anyway, anything that was the least helpful because I tried to take something out of every session even, like, the exit strategy’. There was a mixture of unhelpful components that were identified across other participants, including the family tree homework, the resilience and strength section of the final session and how wordy some of the CAT‐GSH manual could be. However, these factors did not seem to have an impact on the perceived helpfulness of the intervention.

##### Providing facilitation choice

Four participants, including one patient and three PWPs, spoke about how CAT‐GSH increased patient choice when the presenting problem was depression. CAT‐GSH was described as a different approach from other GSH and provided and overview of a patient's narrative that was absent from CBT‐GSH. PWP2 explained ‘I think it's always good to have again like I say just different kind of interventions, erm, at low intensity and just doing IAPT in general as well’.

#### Ethicality

Ethicality is the extent to which CAT‐GSH for depression had a good fit with an individual's values. This theme was supported by all 14 participants and included subthemes: valuing mental health and valuing insight.

##### Valuing mental health

Both PWPs and patients explained they valued mental health and felt good mental health was important for them and wider society. Improved mental health for patients enabled them to attend work, look after their children and improved their relationships. CAT‐GSH for depression fitted with this value because patients perceived the intervention as a GSH that was investing and invested in improving their well‐being. PWPs had similar perspectives and so noted that CAT‐GSH was in line with their professional and personal helping values. For example, PWP 2 stated ‘It's always good to have, erm, yeah healthy mental wellbeing because it helps you in, you know, in your job, friends, family every day it helps you so it's very important to me’.

##### Valuing insight

Understanding distress was important for participants, regardless as to whether mood then actually improved. CAT‐GSH aligned with this value because the approach encouraged patients to ‘get to the root’ of their depression and helped them understand the link between their past and current relationships. The PWPs agreed that patients valued the improved insight that CAT‐GSH for depression facilitated. For example, PWP3 explained ‘Probably that kind of realisation, for a lot, that realisation for a lot of clients where they see how they've maybe adopted some of those past kind of relationship roles and they're kind of reflecting that towards themselves now’.

### Self‐efficacy

Self‐efficacy described the participants confidence they could perform the behaviours required to participate in CAT‐GSH for depression. Self‐efficacy was supported by 13 participants (i.e., 10 patients and 3 PWPs). Confidence levels tended to fluctuate and depended on the support patients received from the PWPs, their understanding of the task and practice.

#### Confidence fluctuation

The confidence of patients to complete the tasks of CAT‐GSH for depression fluctuated and this was associated with motivation, life events and understanding. Patient 4 explained ‘Some I found really easy, some I found difficult, some I thought oh I think I'm doing this right but I'm not sure’. One patient did not feel confident in engaging in tasks due to their poor eyesight. However, PWPs did not report any patients struggling with self‐efficacy, with one patient agreeing that they felt confident in engaging in all the tasks of CAT‐GSH, even on tough days. Regarding PWPs self‐efficacy for facilitation, two PWPs reported that they either did not feel confident in facilitating CAT‐GSH or that their confidence developed with experience.

#### Practice, understanding and support

Of the patients who experienced their confidence fluctuate, they reported that improving their understanding of the task, PWP support and practising improved their confidence. PWPs supported the patient's confidence by explaining the concepts of the intervention and providing emotional support. ‘So, when once we'd sort of discussed the blocks I were putting in me own way, erm, it did become quite easy to overcome and every time I come back to that obstacle I have me tools in place now to overcome it’ (Patient 2).

## DISCUSSION

This study piloted CAT‐GSH for depression within an NHS TT service to begin the process of building a depression‐specific evidence base that mirrors the evaluation pipeline that has been achieved with the anxiety version. This study therefore utilized a practice‐based and mixed methods case series design to initially explore acceptability, define the recovery rates achieved and assess the durability of treatment changes at follow‐up. CAT‐GSH was generally competently delivered, and this mirrors the anxiety CAT‐GSH evidence base (Power et al., 2024). The study had an acceptability target of 10 fully treated cases, and this was achieved after treating just 11 depression cases (i.e., one participant dropped out after three sessions of CAT‐GSH). No participants that were offered CAT‐GSH failed to attend for treatment (i.e., the treatment refusal rate was zero). In the completer sample, all 10 patients attended all six sessions of CAT‐GSH and tended to maintain their depression gains at follow‐up. The shape of the response curve on the PHQ‐9 would suggest that most change occurred in session 5 and 6. This would suggest a phase‐specific effect, as this is the revision stage of CAT‐GSH where change is being implemented based on reformulation and recognition (Ryle & Kerr, 2020).

The acceptability outcomes for the current study should be seen in the context of the evidence that treatment refusal for CBT‐GSH and early dropout during CBT‐GSH has been identified as a major problem for TT services (i.e., 29% never access a single GSH session, 54% dropout early and do not receive an adequate dose and average session attendance is 3.69 sessions; Gonzalez Salas Duhne et al., 2022). In the CAT‐GSH for anxiety evidence base, there is evidence of differentially better acceptability (Kellett et al., 2023), and this is mirrored in a meta‐analysis of the high‐intensity version of CAT (Simmonds‐Buckley et al., 2022). In terms of case‐by‐case outcomes, 7/10 (i.e., 70%) people who completed all six sessions were categorized as ‘reliably recovered’ and this exceeds the TT service target of 48%, albeit in a small sample. Outcomes were comparable to other studies exploring GSH, as demonstrated in the benchmarking comparison exercise (Green et al., 2014; Kellett et al., 2023; Meadows & Kellett, 2017). The durability of the CAT‐GSH indexed by the stable follow‐up outcome scores should be seen in the context of the high relapse rate that has been observed for CBT‐GSH (Ali et al., 2017).

Qualitatively, the findings are also comparable to the CAT literature in terms of the important role of the mid‐stream recognition phase and CAT‐GSH for depression adheres to the three‐phase CAT structure (Meadows & Kellett, 2017). Participants spoke about having increased awareness of their depression relational roles and associated patterns. Increased insight because of CAT has previously been associated with better management of depression (Rayner et al., 2011). Insight is developed through self‐recognition, between‐session work and also in‐session feedback from therapists (Sandhu et al., 2017). CAT‐GSH for depression enables better recognition through the regular between‐session homework task of noticing when key problematic roles and patterns are being enacted. The recognition phase of CAT‐GSH is one of the defining features of the approach that sets it apart from CBT‐GSH, as is the ‘past‐present’ focus (Kellett et al., 2023). This type of work was seen as a necessary precursor of change by patient participants. When considering aspects of CAT‐GSH that were less acceptable, patients tended to want a longer intervention, while PWPs suggested changes to content. For example, patients reported wanting more sessions or less clinical environments, while the PWPs reported the family tree and the strength/resilience sections could be improved. Wanting more flexibility with frequency, length or number of sessions is commonly reported in GSH literature (Krebber et al., 2016; Wray et al., 2022). Delivery of more sessions at increased intervals has been previously associated with improved outcomes within NHS TT services (Saunders et al., 2020). Providing negotiated additional sessions to help patients understand the current content of CAT‐GSH, with flexible intervals, could have improved outcomes.

### Limitations and future directions

The major limitations of this study were the lack of both diagnostic certainty and randomisation, the brief follow‐up period in the small sample and the lack of joint coding of interviews. Future studies would benefit from interviewing patients that drop out of CAT‐GSH alongside using validated measures of acceptability. Longer follow‐ups in GSH studies are needed. This evidence supports progressing onto a larger scale and more controlled evaluation of CAT‐GSH for depression. This could be achieved in two ways: (1) a head‐to‐head randomised control trial of CBT‐GSH versus CAT‐GSH and (2) considering the rate of treatment return (Lorimer et al., 2024) and the ethical dilemma of offering the same intervention again when there is a lack of LI treatment plurality; then, a trial of CAT‐GSH for treatment returners to TT services is also warranted. The mechanism of change for CAT‐GSH for depression needs to be investigated as this has been identified for anxiety (Headley et al., 2024). Furthermore, the burden of CAT‐GSH needs to be better considered, and future evaluations should also include measures of possible negative intervention effects (e.g., the Negative Effects Questionnaire; Rozental et al., 2019).

## CONCLUSION

This first evaluation of CAT‐GSH for depression has provided encouraging evidence that it is an acceptable intervention for PWPs to deliver and patients to receive, which appears to enable reductions to depression that are maintained at follow‐up. CAT‐GSH would appear to offer improved treatment choice within TT services as part of the LI suite, and PWPs seem to enjoy delivering CAT‐GSH. Owen et al. (2021) have identified PWPs as an at‐risk group for stress and burnout, and CAT‐GSH offers useful variability to the PWP clinical role. Caution should still be applied to the current findings due to the natural methodological flaws of any practice‐based case series design. More controlled research is needed to overcome the limitations of the current study and to complete the next phase of developing the CAT‐GSH for depression evidence base.

## AUTHOR CONTRIBUTIONS

**Rebecca Kelly:** Investigation; methodology; formal analysis; project administration; data curation; writing – original draft; writing – review and editing. **Stephen Kellett:** Conceptualization; investigation; writing – original draft; methodology; writing – review and editing; project administration; supervision. **Mel Simmonds‐Buckley:** Writing – original draft; writing – review and editing; supervision. **Niall Power:** Formal analysis; writing – original draft; writing – review and editing.

## FUNDING INFORMATION

None.

## CONFLICT OF INTEREST STATEMENT

None.

## Supporting information


Appendix S1.

## Data Availability

Data that support the findings of this study are available from the corresponding author upon request.
