# Supplementary material for: Cognitive analytic therapy‐guided self‐help for depression: A mixed methods evaluation
Source: Psychol Psychother. 2025 Aug 18;99(1):97–112. doi: 10.1111/papt.70008 (PMC12905521; doi:10.1111/papt.70008)
Supplement: Supplementary file 1 — Appendix S1. [file PAPT-99-97-s001.docx]

**Supplementary materials**

**Quality assurance, reflexivity and ontological position**

The lead researcher utilised supervision to discuss decisions and resolve uncertainties. Triangulation of different sources was also used for quality assurance (Korstjens & Moser, 2018). A reflexivity diary was kept outlining the lead researcher’s assumptions, preconceptions, and how this affected research decisions (Korstjens & Moser, 2018). The non-linear process included continuously reviewing, adapting, and outcoming. The diary included, but was not limited to, contemplating inter-personal aspects, personal reflections, epistemological considerations, organisational and service implications, ethical dilemmas, and conflicting interests. A critical realist-informed perspective helped develop and implement this research. It was assumed the reality of CAT-GSH was impacted by the researchers, facilitators, and patients’ perspectives alongside broader social narratives and systems (Braun & Clarke, 2006). A subjective epistemological position assumed that the measurement of reality was impacted by personal beliefs, interpretations, and measurement constructs. For example, Framework Analysis (Ritchie & Spencer, 1994), was used to analyse the data as it aims to understand what occurs behind the descriptions of the phenomena by using both research and data driven processes. Mercier et al., (2023) argues this aligns with a critical realist perspective which holds the “tensions” between perceptions of reality and theoretical descriptions.

**Online Table 1;** Range and median of CAT-GSH training satisfaction scores

|  | Range | Median |
| --- | --- | --- |
| 1) I feel sufficiently knowledgeable about CAT guided self-help at a theoretical level. | 8 – 10 | 8 |
| 2) I feel I sufficiently understand the three components of CAT-GSH. | 8 | 8 |
| 3) I feel sufficiently knowledgeable about how CAT-GSH differs from CBT-GSH. | 9 | 9 |
| 4) I feel sufficiently confident in implementing CAT-GSH. | 6 – 8 | 7 |
| 5) I am generally satisfied with the training provided on CAT-GSH. | 9 | 9 |
| 6) The training materials were presented in an engaging and understandable way. | 9 – 10 | 9.5 |
| 7) The trainers were encouraging and enthusiastic. | 10 | 10 |

| 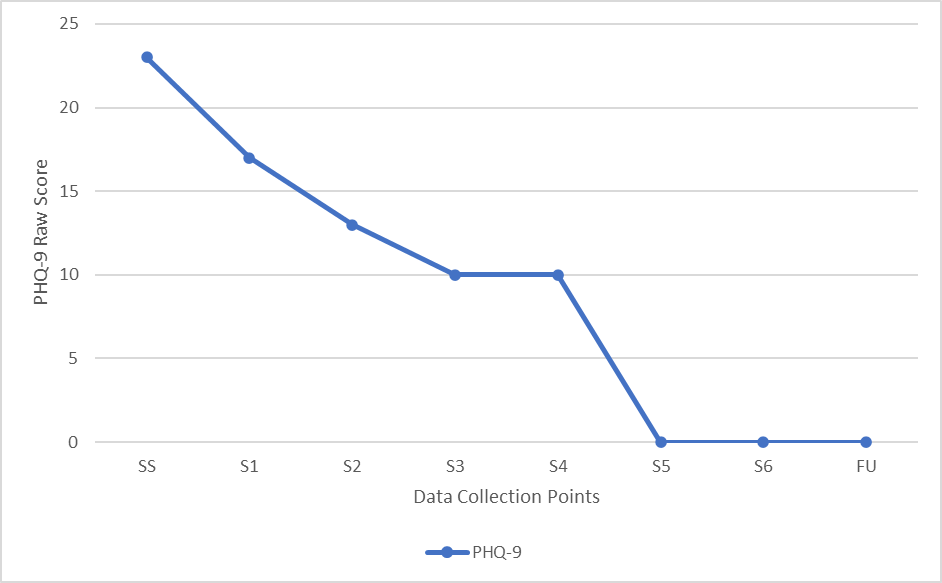 | 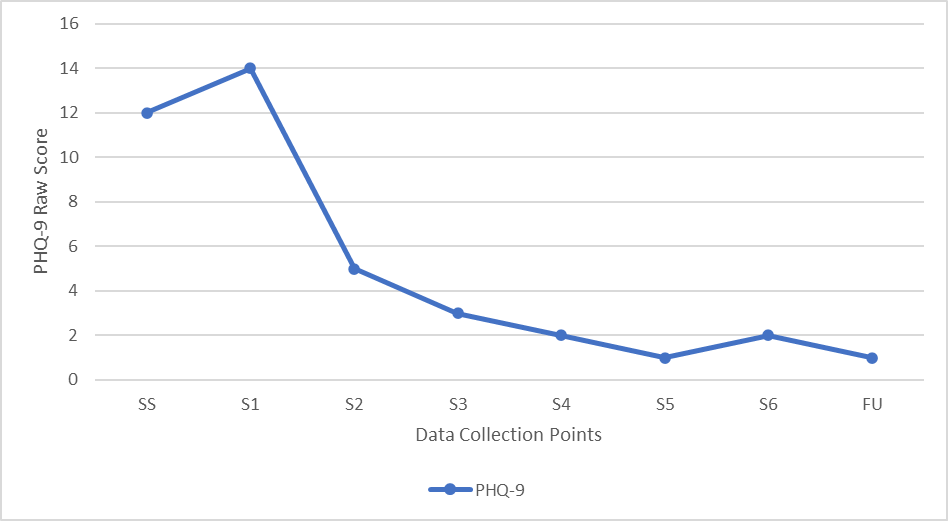 |
| --- | --- |
| 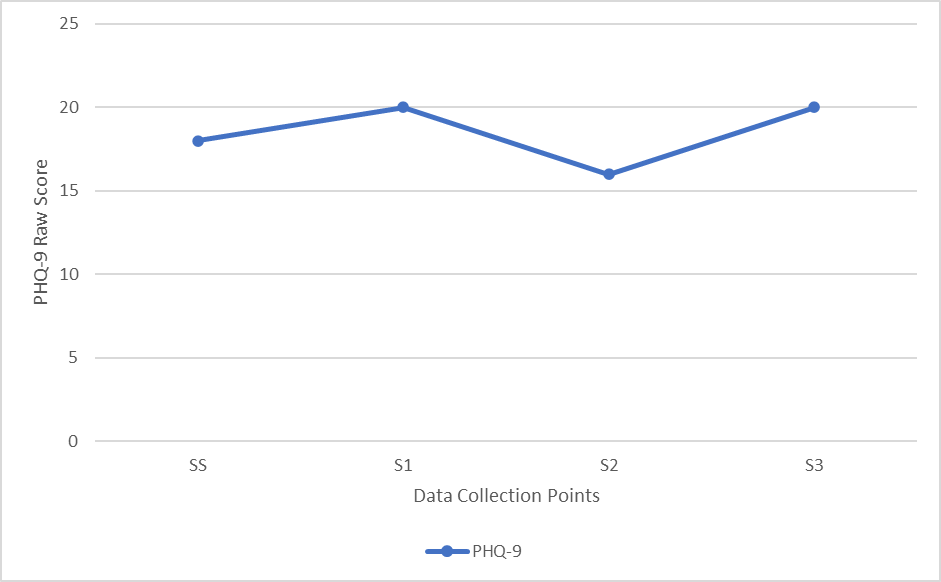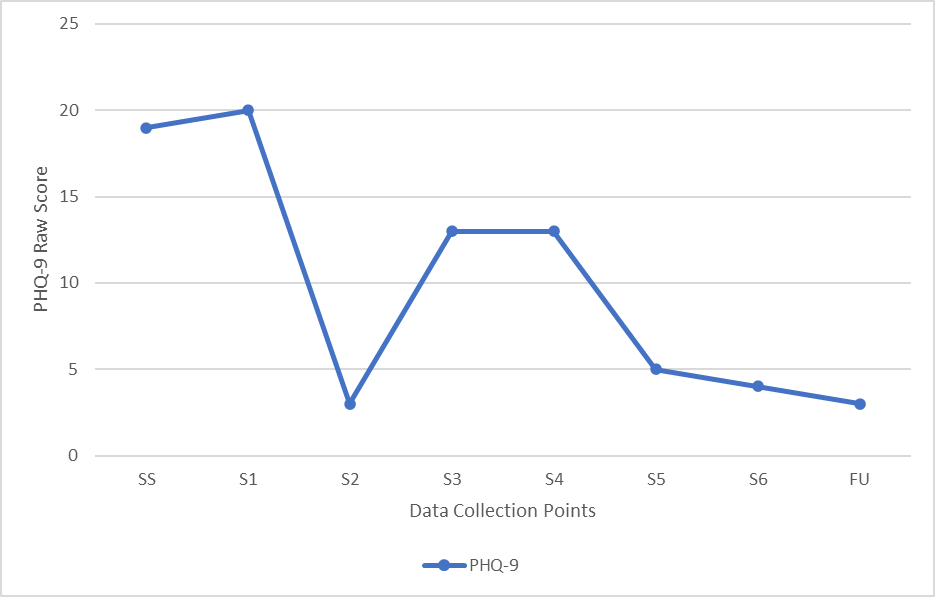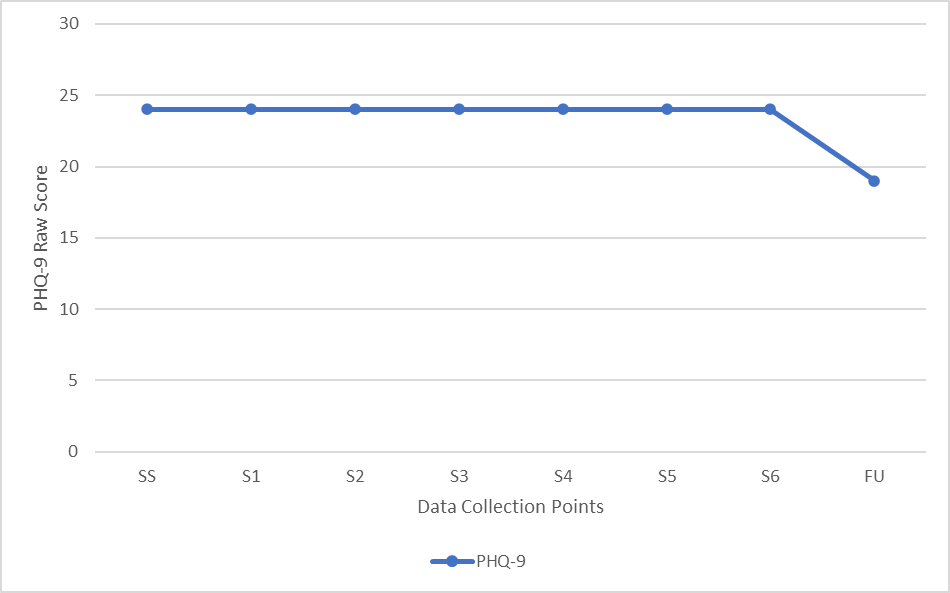 | 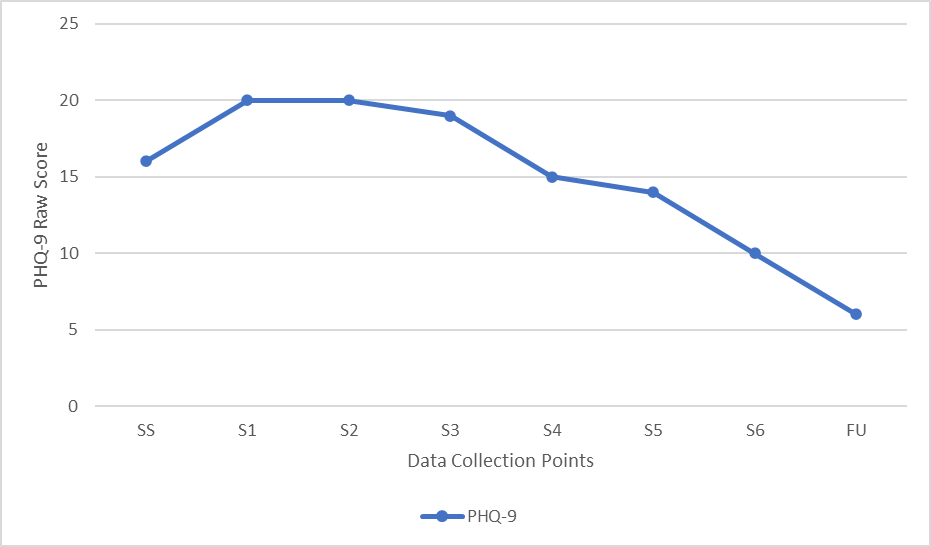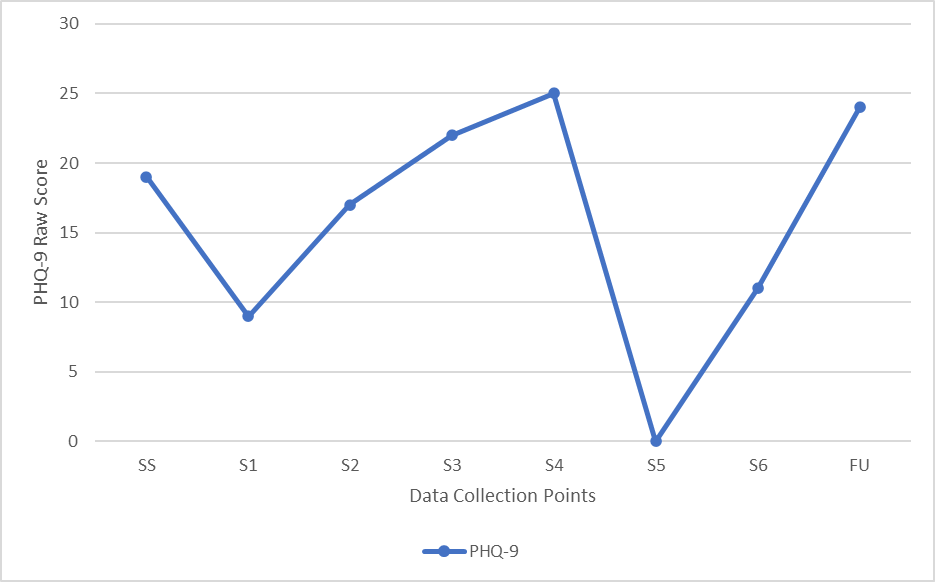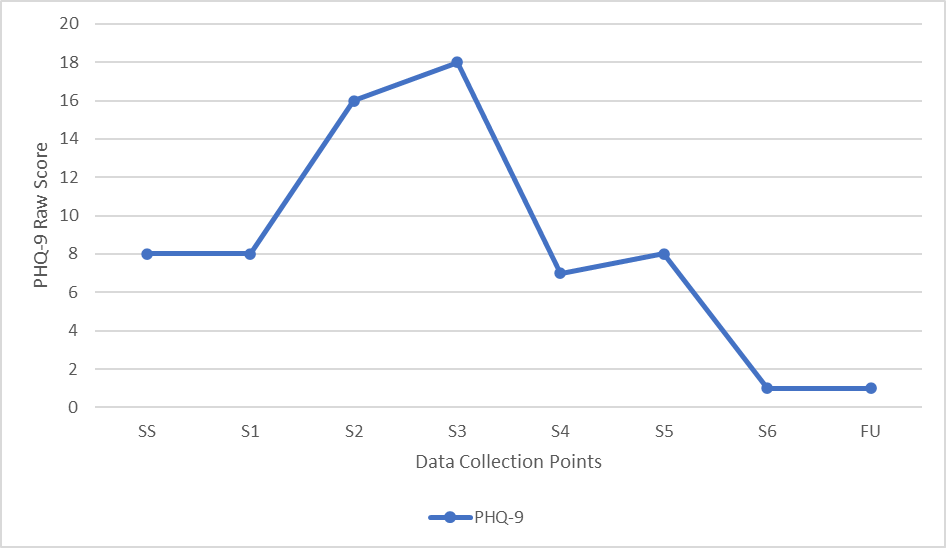 |
| 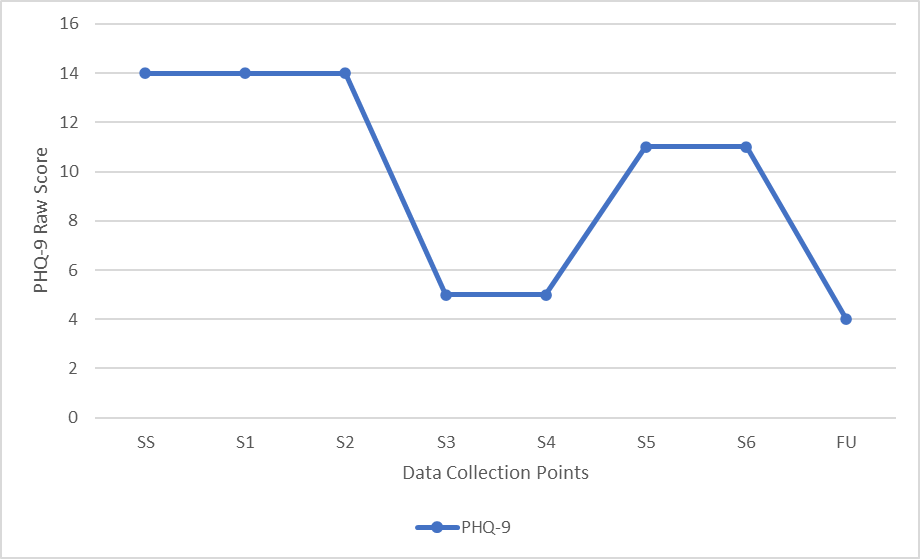 | 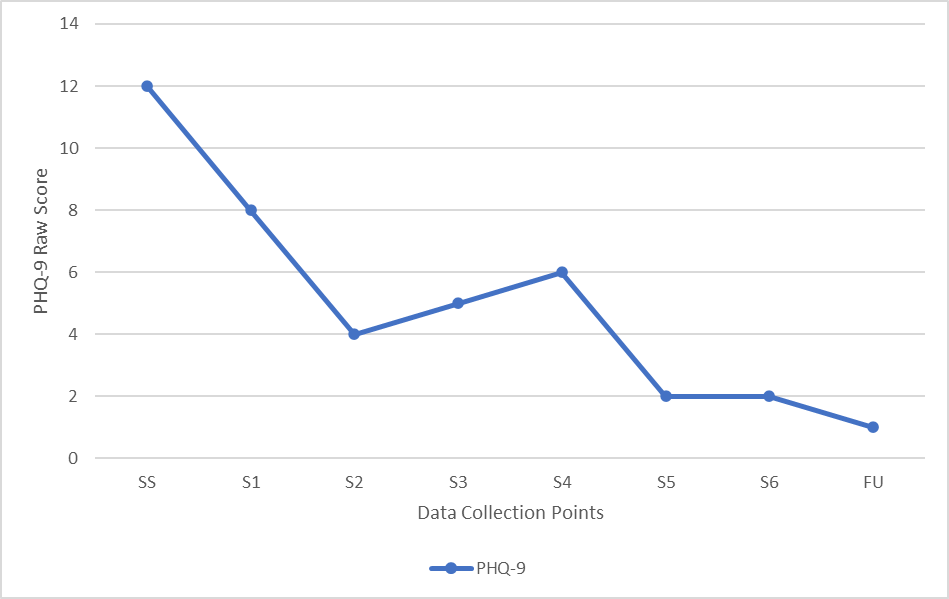 |
| 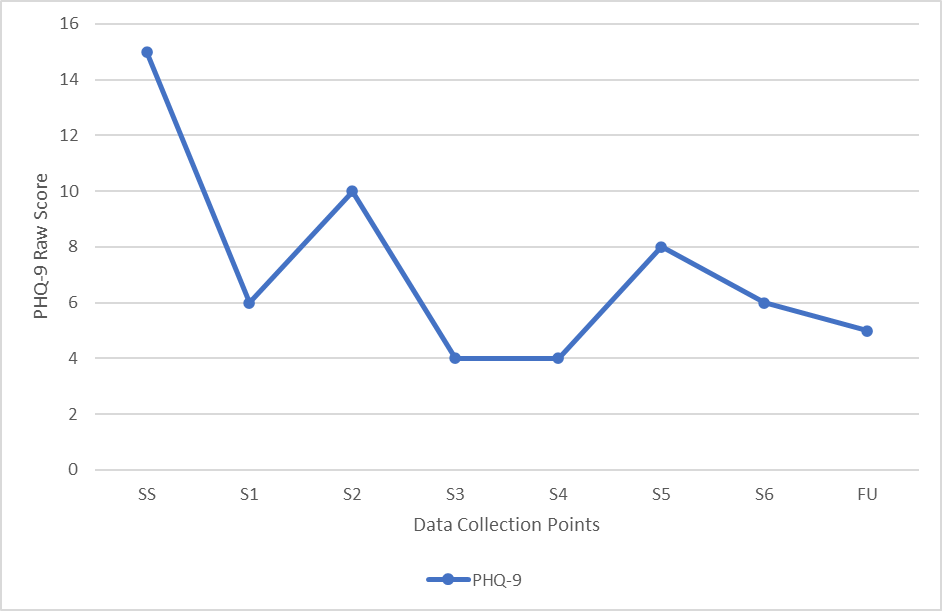 |  |
|  |  |
|  |  |

**Online supplementary Figure 2**; case by case PHQ-9 plots

**Online Table 2*;*** Raw scores of screening (S), termination (T) and follow-up (FU); change in scores; RCI, CSC and PHQ-9 classifications.

|  | Screen | Term | Follow-up | Change Score (B-T) | RCI | CSC | Chance Score (B-FU) | Reliable change | Clinical change | Outcome classification at follow-up |
| --- | --- | --- | --- | --- | --- | --- | --- | --- | --- | --- |
| Participant 1  PHQ-9  GAD-7 | 23  16 | 0  0 | 0  0 | -23  -23 | Yes  Yes | Yes  Yes | -23  -23 | Yes  Yes | Yes  Yes | Reliable Recovery |
| Participant 2  PHQ-9  GAD-7 | 12  4 | 2  2 | 1  2 | -10  -2 | Yes  No | Yes  No | -11  -2 | Yes  No | Yes  No | Reliable Recovery |
| Participant 3  PHQ-9  GAD-7 | 24  24 | 24  16 | 19  16 | 0  -8 | No  Yes | No  No | -5  -8 | No  Yes | No  No | Uncertain Change |
| Participant 4  PHQ-9  GAD-7 | 8  6 | 1  2 | 1  2 | -7  -4 | Yes  Yes | No  No | -7  -4 | Yes  Yes | No  No | Reliable Improvement |
| Participant 5  PHQ-9  GAD-7 | 18  16 | 20  20 |  | +2  +4 | No  No | No  No |  |  |  | Uncertain Change (for termination) |
| Participant 6  PHQ-9  GAD-7 | 16  16 | 10  9 | 6  4 | -6  -7 | Yes  Yes | No  No | -10  -12 | Yes  Yes | Yes  Yes | Reliable Recovery |
| Participant 7  PHQ-9  GAD-7 | 14  9 | 11  11 | 4  3 | -3  +2 | No  No | No  No | -10  -6 | Yes  Yes | Yes  Yes | Reliable Recovery |
| Participant 8  PHQ-9  GAD-7 | 19  11 | 11  14 | 24  21 | -8  +3 | Yes  No | No  No | +5  +10 | No  No | No  No | Uncertain Change |
| Participant 9  PHQ-9  GAD-7 | 19  5 | 4  1 | 3  0 | -15  -4 | Yes  Yes | Yes  No | -16  -5 | Yes  Yes | Yes  No | Reliable Recovery |
| Participant 10  PHQ-9  GAD-7 | 12  11 | 2  7 | 1  2 | -10  -4 | Yes  Yes | Yes  Yes | -11  -9 | Yes  Yes | Yes  Yes | Reliable Recovery |
| Participant 11  PHQ-9  GAD-7 | 15  14 | 6  2 | 5  4 | -9  -12 | Yes  Yes | Yes  Yes | -10  -10 | Yes  Yes | Yes  Yes | Reliable Recovery |

*Note.* Participant 5 screening was compared to session three termination.
